# Supplementary material for: Near-Edge X-Ray Absorption Fine-Structure Spectra and Specific Dissociation of Phe-Gly and Gly-Phe
Source: Int J Mol Sci. 2025 Mar 11;26(6):2515. doi: 10.3390/ijms26062515 (PMC11942449; doi:10.3390/ijms26062515)
Supplement: Supplementary file 1 [file ijms-26-02515-s001.zip › ijms-3469995-supplementary.pdf]

Near-Edge X-Ray Absorption Fine-Structure Spectra and Specific Dissociation of Phe-Gly and Gly-Phe

Tse-Fu Shen [1], Yu-Ju Chiang [1], Yi-Shiue Lin [1], Chen-Lin Liu [1,2,*], Yu-Chiao Wang [3], Kuan-Yi Chou [3], Cheng-Cheng Tsai [3] and Wei-Ping Hu [3,*]

1 Scientific Research Division, National Synchrotron Radiation Research Center, Hsinchu 300092, Taiwan

2 International PhD Program for Science, National Sun Yat-sen University, Kaohsiung 80424, Taiwan

3 Department of Chemistry and Biochemistry, National Chung Cheng University, Chia-Yi 62102, Taiwan

* Correspondence: liu.cl@nsrrc.org.tw (C.-L.L.); chewph@ccu.edu.tw (W.-P.H.)

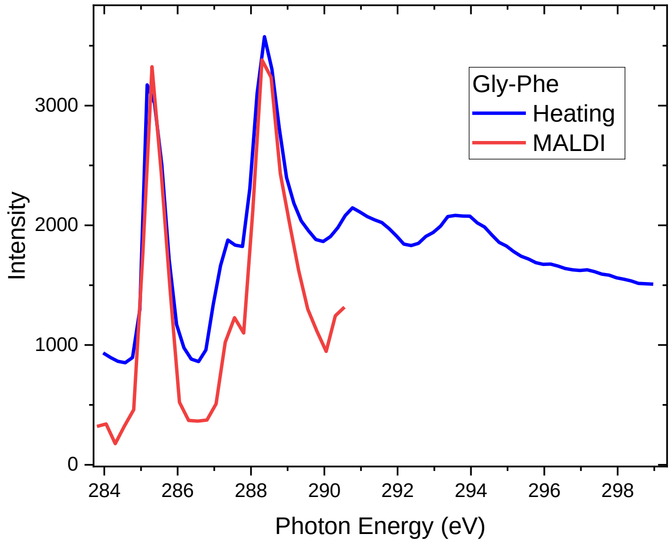

Figure S1. Total-ion-yield mode NEXAFS spectra of Gly-Phe at the carbon K-edge, comparing results obtained using MALDI and heating methods.

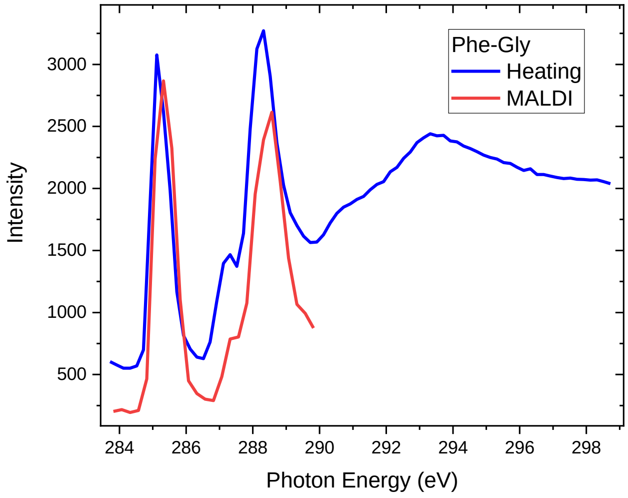

Figure S2. Total-ion-yield mode NEXAFS spectra of Phe-Gly at the carbon K-edge, comparing results obtained using MALDI and heating methods.

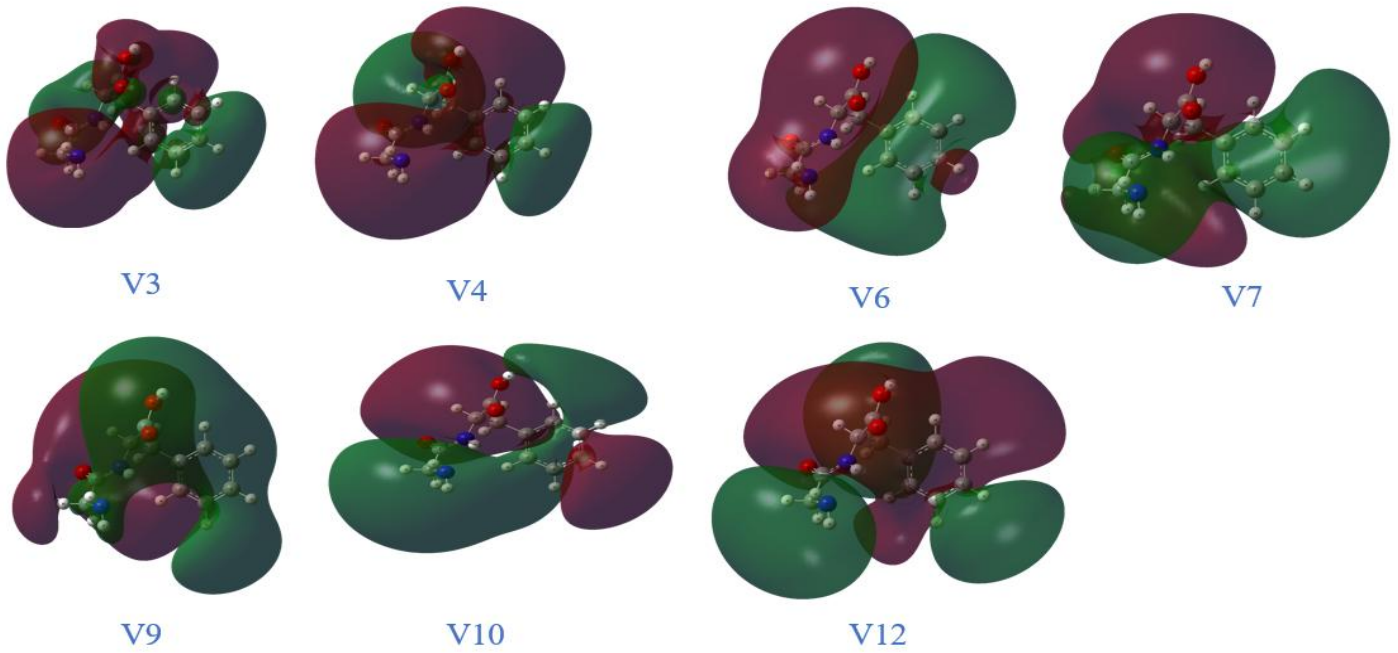

Figure S3. A complete set of core-excitation destination orbitals of Gly-Phe derived from theoretical calculations.

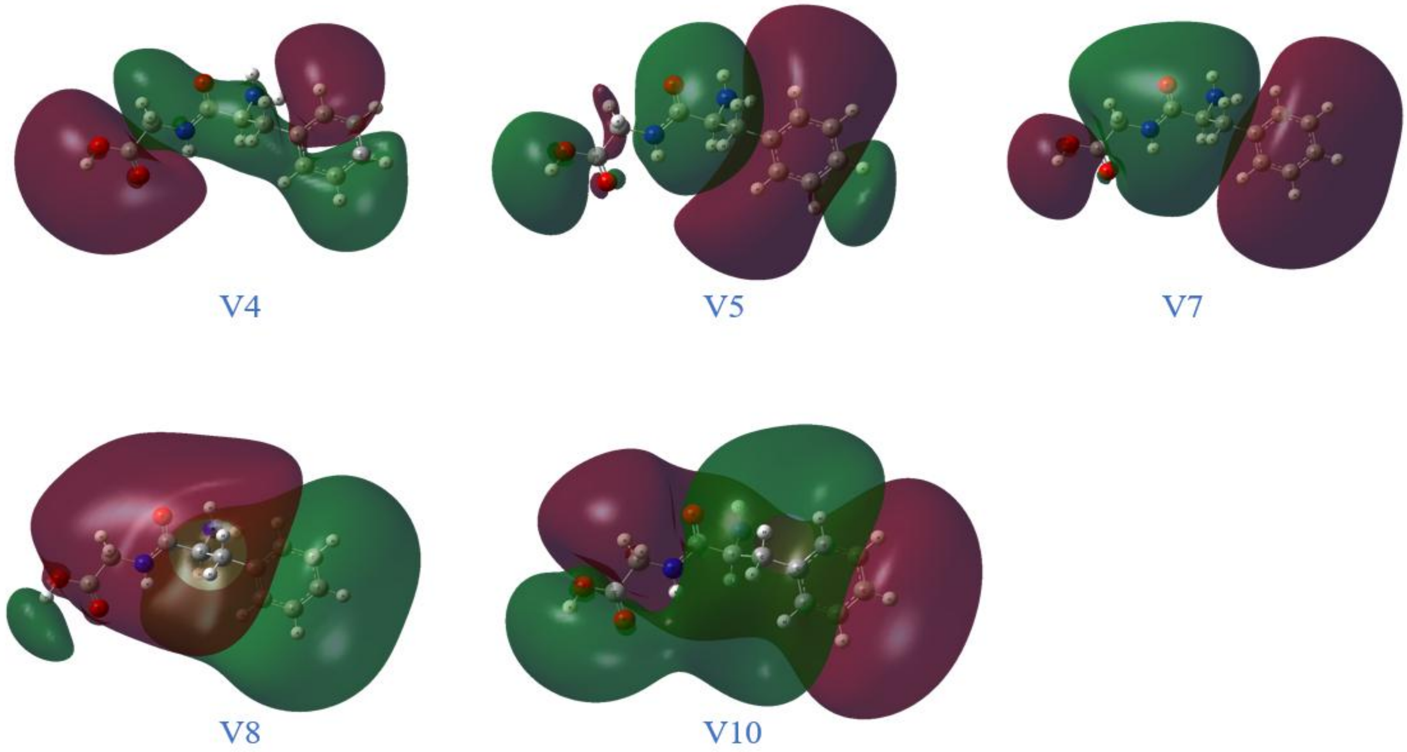

Figure S4. A complete set of core-excitation destination orbitals of Phe-Gly derived from theoretical calculations.
